# Supramaximal Horizontal Rectus Recession–Resection Surgery for Complete Unilateral Abducens Nerve Palsy

**DOI:** 10.3389/fmed.2021.795665

**Published:** 2022-02-22

**Authors:** Zhonghao Wang, Licheng Fu, Tao Shen, Xuan Qiu, Xinping Yu, Huangxuan Shen, Jianhua Yan

**Affiliations:** State Key Laboratory of Ophthalmology, Zhongshan Ophthalmic Center, Sun Yat-sen University, Guangzhou, China

**Keywords:** paralytic strabismus, rectus recession-resection, complete abducens palsy, surgical dosage-effect, surgical outcome

## Abstract

**Purpose:**

To review the surgical procedures and outcomes of supramaximal horizontal rectus recession–resection surgery for abduction deficiency and esotropia resulting from complete unilateral abducens nerve palsy.

**Methods:**

A total of 36 consecutive cases diagnosed as complete abducens nerve palsy, receiving supramaximal medial rectus recession (8.5 ± 1.4 mm, range: 6–10) combined with a supramaximal lateral rectus resection (11.1 ± 1.7 mm, range: 8–14) as performed over the period from 2017 to 2020, were reviewed retrospectively. All surgeries were performed by a single surgeon. Pre- and post-operative ocular motility, ocular alignment, forced duction test, binocular vision, abnormal head posture, and surgical complications were assessed.

**Results:**

Of these 36 cases, 23 (63.8%) were followed up for greater than 2 months (Mean ± SD = 8.4 ± 6.0, range: 2–24) after surgery and the collected data was presented. Mean ± SD age of these patients was 41.7 ± 14.4 (range: 12–67) years with 73.9% being female. Trauma (52.2%, 12/23) and cerebral lesions (21.7%, 5/23) were the primary etiologies for this condition. Esodeviation in primary position improved from 55.5 ± 27.2 prism diopters (PD) (range: +25 to +123) to 0.04 ± 7.3 PD (range: −18 to +12) as assessed on their last visit. Pre-operative abduction deficits of −5.6 ± 1.0 (range: −8 to −4) reduced to −2.4 ± 1.4 (range: −4 to 0) post-operatively. The mean dose-effect coefficient of 2.80 ± 1.20 PD/mm (range: 1.07–6.05) was positively correlated with pre-operative esodeviation. Rates of overcorrection and ortho were 69.6 and 26.1%, respectively, on the first day after surgery, while on their last visit the respective levels were 4.3 and 82.6%.

**Conclusion:**

Supramaximal horizontal rectus recession–resection surgery is an effective treatment method for complete abduction deficiency. The dose-effect was positively correlated with pre-operative esodeviation. Overcorrection on the first day post-operatively is required for a long-term satisfactory surgical outcome.

## Introduction

The abducens nerve represents the most vulnerable ocular motor nerve, with abducens nerve palsy resulting in esodeviation and horizontal diplopia. Such a condition often involves a compensatory head turn to minimize or eliminate the diplopia. If the esodeviation is permanent and remains stable for more than six months, strabismus surgery will be required to decrease or eliminate head turn, center and expand the single binocular visual fields, and increase abduction while preserving adduction (1).

Horizontal rectus recession–resection without transposition of the vertical rectus can correct this deviation and improve the abduction in cases with partial paralysis (2, 3). However, this recession–resection surgery is not recommended for patients with complete lateral rectus paralysis, due to the lack of a successful outcome resulting from regression of ocular alignment and failure to restore abduction function (4).

A complete abduction deficiency always involves treatment with various surgical techniques consisting of vertical rectus transposition (VRT), including the Hummelsheim or Jensen procedure (4–8) and isolated superior rectus transposition (SRT) (9). However, many disadvantages are associated with VRT, such as the risk of anterior segment ischemia syndrome and new vertical/torsional deviations, which are often difficult to manage even with reoperation (10, 11). In addition, the effects of transposition depend on the tension of the transposed muscle and may not effectively increase the abduction function of the affected eye. In this way, the velocity of rotation into the paretic field is fairly slow and may not be useful when rapid pursuit or saccadic movements are required (12).

Lee (13) reported that maximal medial rectus muscle recession and lateral rectus muscle resection, i.e., the Hummelsheim procedure and the Jensen procedure, may be equally effective in the surgical treatment of complete and partial lateral rectus muscle paralysis. Bagheri (14) suggested that the horizontal rectus resection/recession procedure may also be effective in the treatment of complete abducens palsy. Since 2017, we have employed a supramaximal horizontal rectus recession–resection procedure for the treatment of complete lateral rectus muscle paralysis in our center. We found that this surgical approach proved effective, not only in correcting the horizontal deviation in the primary position but also in improving the abduction function. The purpose of this report is to review the outcomes of supramaximal horizontal rectus recession–resection surgery for complete unilateral abduction deficiency and esodeviation as resulting from an acquired complete sixth nerve palsy.

## Patients and Methods

### Patients

Institutional approval (Approval No: 2021KYPJ050) for this study was obtained from the Research Ethics Board of the Zhongshan Ophthalmic Center (ZOC), Sun Yat-sen University, China. All procedures were performed in accordance with the 1964 Declaration of Helsinki. A retrospective analysis was conducted on all consecutive patients with complete abduction deficiency resulting from acquired sixth nerve palsy, who underwent supramaximal horizontal rectus recession–resection surgery in the ZOC during the period from January 2017 to December 2020. All patients were surgically treated by the corresponding author (JH Yan). Written informed consent to participate in this study was obtained before surgery from all patients or from the parents of those younger than 18 years of age.

Complete unilateral abducens palsy was defined as an acquired paralytic esotropia with abduction failing to cross the middle line during monocular gaze (abduction < -4). A force generate test was conducted to confirm the absence of active contracture of the lateral rectus, while forced duction tests (FDTs) were employed to rule out any restrictive elements. The exclusion criteria included previous strabismus surgery or botulinum toxin injections, orbital fracture related to mechanical limitations as confirmed by imaging and/or FDT results, and any other combination of cranial nerve palsies. Surgery was planned when the angle of esotropia was stable and >15 PD after a minimal period of 6 months of conservative treatment.

### Data Collection

All patients underwent a complete ophthalmic evaluation. Data collected included sex, age, etiology, pre- and post-operative deviations in the primary and six diagnostic positions of gaze, FDT, ocular motility, abnormal head posture, binocular vision, and recordings of vertical deviation or any other complications. Deviations in the primary position at a distance (6 m) were measured using the simultaneous prism and cover test, with the prism over the paretic eye. Patients with poor visual acuity were tested using the Krimsky test. Prisms were split between the two eyes in patients with >50 PD esodeviation. Duction was recorded on a scale of 0 to −8, with 0 indicating full duction, −4 indicating eye rotation only to the midline, and −8 indicating that the eye was fixed in an extreme adducted position (9). Duction range of the paretic eye was quantitatively evaluated by the sum of abduction and adduction scales with 8 as the full duction range. Fusion and stereopsis were determined with the use of the worth-4-dot, synoptophore, and random dot stereo tests. Patients were followed up on day 1, after 2 months, and then every 3 to 6 months after surgery. Alignment success was defined as a deviation within 10 PD in primary position, as determined on their last visit.

### Surgical Technique

All surgeries were performed through fornix conjunctival incisions while patients were under general anesthesia. The forced duction test was routinely performed during surgery. Supramaximal recession–resection dosage of the horizontal rectus was performed according to the horizontal deviation (Table 1). The medial rectus was recessed 6–10 mm using the “hang-back” technique with 6–0 polyglactin suture, while lateral rectus was resected 8–14 mm.

**Table 1:** Surgical dosage form of supramaximal R-R operation.

**Deviation** **(PD)**	**MR Rec** **(mm)**	**LR Res** **(mm)**
25	6	7
35	7	8
45	8	10
50	9	11
55	10	12
>55	10	13–14

*PD, Prism diopter; MR, Medial rectus; LR, Lateral rectus; Rec, Recession; Res, Resection*.

### Statistical Analysis

Statistical analysis was performed using the SPSS version 23.0 program (SPSS Inc., Chicago, IL, USA). Quantitative data were analyzed using independent sample and paired *t*-tests. Qualitative classification data were analyzed using the chi-square test. Normality of the data was tested using Kolmogorv–Smirnov (K-S) test and Q-Q plots, and homogeneity of variance was assessed with the use of Levene's test. paired T-test or independent-sample T-test were performed for variables data which conforming to normal distribution. Non-parametric statistical tests were used for the data which did not conform to the normal distribution, i.e., paired samples were based on Wilcoxon test, and independent samples were based on Mann–Whitney *U*-test. Multiple linear regression was used to analyze the independent factors of surgical dosage effect. A *P* value < 0.05 was required for results to be considered as statistically significant.

## Results

A total of 36 consecutive patients, who were diagnosed with complete abducens palsy resulting from acquired sixth nerve paresis and underwent supramaximal horizontal rectus recession–resection surgery, were reviewed. Of these 36 cases, 23 (63.9%) were followed up for >2 months post-operatively. Data presented represents that from the 23 cases with the >2 months follow-up periods. Ages ranged from 12 to 67 years (Mean ± SD = 41.7 ± 14.4), with 73.9.0% (17/23) being females. The median course of the sixth nerve palsy was 18 months (range: 8–720). Trauma was considered as the main cause of this condition in 52.2% (12/23) of the cases, while 21.7% (5/23) had cranial lesions, 8.7% (2/23) had vascular lesions, and 17.4% (4/23) were of unknown etiology. Surgery dosages were 8.5 ± 1.4 mm (range: 6–10) for medial rectus recession and 11.1 ± 1.7 mm (range: 8–11) for lateral rectus resection. There were no statistically significant differences in demographics, clinical characteristics, or surgical dosage among the 23 patients with the >2 months follow-up periods and the 13 lost cases, except that this latter group was younger in age (Table 2). The mean follow-up time was 8.4 ± 6.0 months (range: 2–24).

**Table 2:** General demographic and clincial features of patients with sixth nerve palsy.

	**Total cases**	**Included cases**	**Failure to visit cases**	***P***
	**(*n* = 36)**	**(*n* = 23)**	**(*n* = 13)**	
Age (range), year	37.8 ± 14.9 (12–67)	41.7 ± 14.4 (12–67)	31.0 ± 13.7 (14–62)	0.035a^*^
Sex, male/female	9/27	6/17	3/10	0.84b
Laterality, right/left	17/19	11/12	6/7	0.92b
Course (median, range), months	24 (8–720)	18 (8–720)	36 (12–444)	0.24c
Etiology				0.81b
Trauma, *n* (%)	17 (47.2)	12 (52.2)	5 (38.5)	
Cerebral lesions, *n* (%)	9 (25)	5 (21.7)	4 (30.8)	
Microvascular lesions, *n* (%)	4 (11.1)	2 (8.7)	2 (15.4)	
Unknwon, *n* (%)	6 (6.7)	4 (17.4)	2 (15.4)	
Deviation (range), PD	59.9 ± 27.9	55.5 ± 27.2 (25–123)	67.7 ± 28.6 (30–113)	0.15c
Abduction (range)	5.44 ± 1.1	−5.6 ± 1.0 (−4–−8)	−5.2 ± 1.2 (−4–−8)	0.34c
Adduction (range)	1.0 ± 0.7 (0–3)	1.0 ± 0.7 (0–2)	1.2 ± 0.9 (0–3)	0.60c
Amount of surgery MR recession (range), mm	8.4 ± 1.4 (5–10)	8.5 ± 1.4 (6–10)	8.1 ± 1.5 (5–10)	0.38c
LR resection (range), mm	11.1 ± 1.8 (7–15)	11.1 ± 1.7 (8–14)	11.2 ± 2.0 (7–15)	0.92c

*PD, Prism diopter; MR, Medial rectus; LR, Lateral rectus; n, Number; a, Based on T test; b, Based on chi-square test; c, Based on Mann-Whiteney U-test; P, P-value;*

* *P-value < 0.05*.

### Ocular Alignment

**Figure 1:**
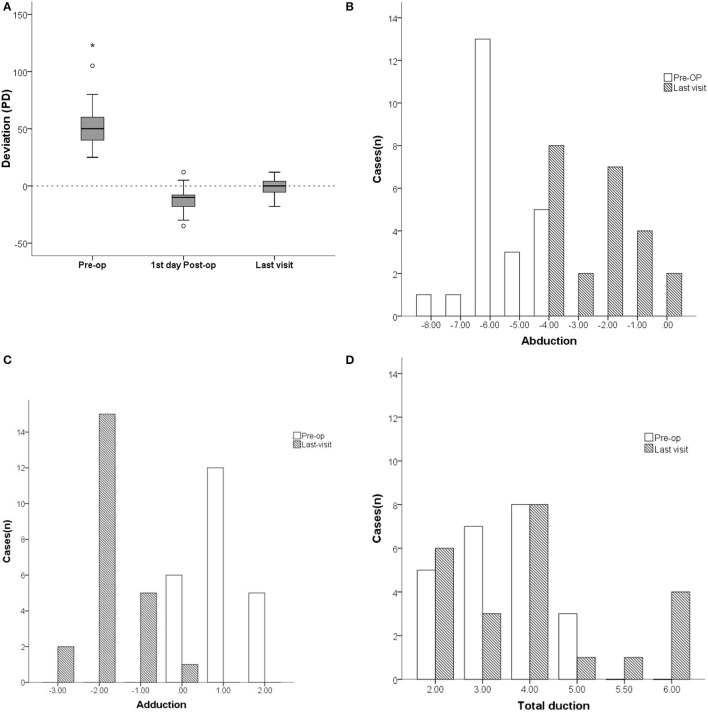
Comparison of deviation (PD) and ocular motility between pre- and postoperation of patients with unilateral complete abducens nerve plasy. **(A)** The mean pre-operative horizontal esodeviation of 55.5 ± 27.2 PD (range: +25 to +123) significantly improved to −11.5 ± 11.0 PD (range: −35 to +12) on the first day after surgery, and further improved to 0.04 ± 7.3 PD (range: −18 to +12) as determined on their last visit. **(B)** The mean abduction function increased form −5.6 ± 1.0 (range: −8 to −4) preoperatively to −2.4 ± 1.4 (range: −4 to 0) on their last visit. **(C)** The mean adductions of the paretic eye decreased from 0.0 ± 0.0 (range: 0 to +2) pre-operatively to a mean deficiency of −1.8 ± 0.7 (range: −3 to 0) on their last visit. **(D)** The mean duction ranges in the paretic eye increased from 2.4 ± 1.0 pre-operatively to 3.8 ± 1.4 on their last visit. In the box plot **(A)**, the value that is more than 1.5 times the distance from the quartile value is the outlier and is represented by “o”, while the value that is more than 2 times is the extreme value and is marked by “*”. Pre-op, pre-operatively.

The mean pre-operative horizontal esodeviation of 55.5 ± 27.2 PD (range: +25 to +123) significantly improved to −11.5 ± 11.0 PD (range: −35 to +12) on the first day after surgery, and further improved to 0.04 ± 7.3 PD (range: −18 to +12) as determined on their last visit (Figure 1A). On the first day after surgery, overcorrection rates and ortho rates were 69.6% and 26.1%, respectively, while they were 4.3% and 82.6%, respectively, on their last visit (Table 3). Vertical deviation was present in 26.1% of pre-operative cases and in 34.7% cases as determined on their last visit, while the mean deviations were 5.0 ± 3.2 PD pre-operatively and 9.6 ± 4.7 PD on their last visit. These differences in mean deviations were not statistically significant.

**Table 3:** Pre- and Postoperative motor/sensory features of patients with unilateral complete abducens nerve palsy.

**Motor and sensory assessment**	**Pre-op (*n* = 23)**	**1st day post-op (*n* = 23)**	**Last visit (*n* = 23)**	**P1**	**P2**
Duction					
Abduction	−5.6 ± 1.0 (−8–−4)	−1.9 ± 1.4 (−4–0)	−2.4 ± 1.4 (−4–0)	0.00^*^	0.057d
Adduction	0	−3.1 ± 0.9 (−4–−1)	−1.8 ± 0.7 (−3–0)	0.00^*^	0.00^*^
Duction range	2.4 ± 1.0	3.0 ± 1.5	3.8 ± 1.4	0.10d	0.10d
Deviation					
Horizontal esodeviation (range), PD	55.5 ± 27.2 (25–123)	−11.5 ± 11.0 (−35–12)	0.04 ± 7.3 (−18–12)	0.00^*^	0.00^*^
Vertical hyperdeviaion (range), PD	5.0 ± 3.2	6.7 ± 2.9	9.6 ± 4.7		
Vertical deviaion, *n* (%)	6 (26.1)	3 (13.0)	8 (34.8)		
Ortho, *n* (%)	–	6 (26.1)	19 (82.6)		
Undercorrection, *n* (%)	–	1 (4.3)	3 (13.1)		
Overcorrection, *n* (%)	–	16 (69.6)	1 (4.3)		
Sensory					
Binocular single vision, *n* (%)	13 (56.5)	14 (60.9)	18 (78.3)		0.18b
Diplopia in the primary position, *n* (%)	17 (73.9)	10 (43.5)	0 (0.0)		0.00b^*^
Abnormal head posture, *n* (%)	8 (34.8)	3 (13.0)	0 (0.0)		0.00b^*^

*Pre-op, Pre-operation; post-op, Post-operation; n, Number; PD, Prism diopter; Ortho, orthotropic; P1, Last visit vs. pre; P2, Last visit vs. 1st day post-op, b, Based on chi-square test; d, Based on Wilcoxon test;*

* *P-value < 0.05*.

### Ocular Motility

The mean pre-operative abduction function of −5.6 ± 1.0 (range: −8 to −4) increased to −1.9 ± 1.4 (range: −4 to 0) on the first day after surgery, and the abduction function significantly improved to a mean abduction deficit of −2.4 ± 1.4 (range: −4 to 0) on their last visit (Figure 1B). Mean adductions of the paretic eye were −3.1 ± 0.9 (range: −4 to −1) on the first day after surgery and presented as a mean deficiency of −1.8 ± 0.7 (range: −3 to 0) on their last visit (Figure 1C). Mean duction ranges in the paretic eye increased from 2.4 ± 1.0 pre-operatively to 3.0 ± 1.5 on the first day after surgery and to 3.8 ± 1.4 on their last visit. No statistically significant difference was found (*P* = 0.10) (Figure 1D; Table 3).

When assessed on their last visit, 15 cases could abduct over the middle line (ABD improved group), while the remaining 8 cases could not abduct over the middle line (ABD unimproved group). Pre-operative abduction deficits were milder in the ABD improved (−5.2 ± 1.0) when compared to the ABD unimproved (−6.30 ± 0.7) group, with pre-operative deviations showing no statistically significant differences between the two groups. In the ABD improved group, 14/15 patients demonstrated a positive FDT test with slight to little resistance, while only 2/8 patients in the ABD unimproved group showed this result (Table 4).

**Table 4:** Comparison of clinical characteristics between patients with improved and unimproved abduction.

**Clinical features**	**ABD improved (*n* = 15)**	**No ABD improved (*n* = 8)**	***P***
Sex (M/F)	10/5	1/7	0.28b
Age (years)	43.9 ± 13.9	37.6 ± 15.2	0.33a
Etiology (trauma/cerebral lesions/others /no)	6/5/1/3	6/0/1/1	0.23b
Deviation	57.2 ± 26.9	52.3 ± 29.2	0.39c
Course (moths)	80.4 ± 180.7	41.4 ± 38.9	0.88c
Abduction	−5.2 ± 1.0	−6.3 ± 0.7	0.04c^*^
Adduction	0.9 ± 0.6	1.1 ± 0.8	0.47c
FDT positive	14/15	2/8	0.01c^*^
MR Rec (mm)	8.2 ± 1.5	9.1 ± 1.0	0.17c
LR Res (mm)	10.9 ± 1.8	11.5 ± 1.5	0.51c
Rec+Res (mm)	19.1 ± 3.0	20.6 ± 2.4	0.36a

*ABD improved, ABD>-4 (over the midline); M, male; F, female; FDT, Forced duction tests; MR, Medial rectus; LR, Lateral rectus; Rec, Recession; Res, Resection; R-R, Recession-Resection, a, Based on T test; b, Based on chi-square test; c, Based on Mann-Whiteney U-test;*

* *P-value < 0.05*.

### Sensory Assessments

Prior to surgery, 56.6% (13/23) of the patients had fusion and stereopsis, 73.9% (17/23) had diplopia in the primary position, and 34.8% (8/23) had abnormal head posture (Table 3). On their last visit, fusion and stereopsis were restored in 78.3% (18/23) of the cases and abnormal head posture and diplopia in the primary position were eliminated in all cases.

### Surgical Dose-Effect of the Supramaximal R-R Surgery

**Figure 2:**
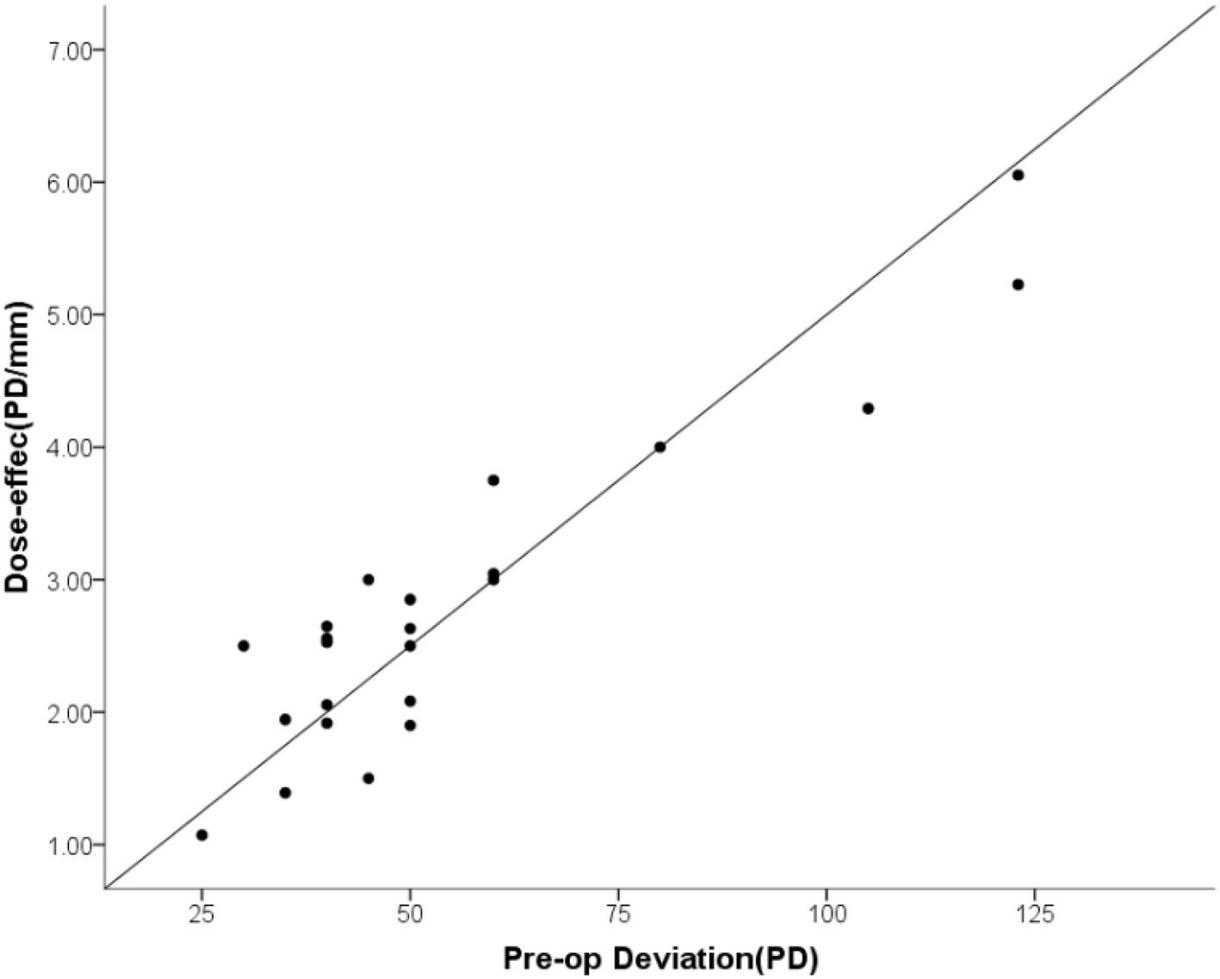
The dose-effect coefficient was linearly related to the pre-operative esodeviation.

The mean surgical dosage of the recession–resection surgery was 19.7 ± 2.9 mm (range: 14 to 24) and the mean dose-effect coefficient was 2.80 ± 1.20 PD/mm (range: 1.07 to 6.05). The dose-effect coefficients were not constant and increased as a function of surgical dosage, with PD/mm values being 1.79 (range: 1.07 to 2.5) for 10–15 mm, 2.56 (range: 1.94 to 6.05) for 16–20 mm, and 2.93 (range: 1.39 to 5.23) for 21–25 mm (Table 5). The dose-effect coefficient was linearly related to the pre-operative esodeviation (Table 6; Figure 2).

**Table 5:** The dose-effect of maximal R-R surgery.

**Rec+Res** **(mm)**	***n***	**Corrected Deviation** **(PD) Median (range)**	**PD/mm Median (range)**
10–15	2	28 (25–30)	1.79 (1.07–2.5)
16–20	9	40 (35–123)	2.56 (1.94–6.05)
21–25	12	50 (35–123)	2.93 (1.39–5.23)

*R-R, Rec-Res = Recession-Resection; PD, Prism diopter*.

**Table 6:** Factors associated with the dose-effect relationship of ultra R-R surgery.

	**Multi-factor analysis**			
	***R***	***p***	***B***	***p***
Sex	0.12	0.59	–	–
Age	0.13	0.55	–	–
Etiology	0.38	0.07	0.62	0.26e
Course	0.57	0.005	0.29	0.49e
Deviation	0.92	0.00	11.07	0.001e^**^
Adduction	0.46	0.03	1.74	0.06e
Abductution	0.223	0.31	–	–
FDT	0.32	0.13	–	–
Binocular vision	0.24	0.27	–	–

*FDT, Forced duction tests;*

** *P-Value < 0.001; e, Based on one-way ANOVA*.

### Typical Case Presentation

#### Case1

**Figure 3:**
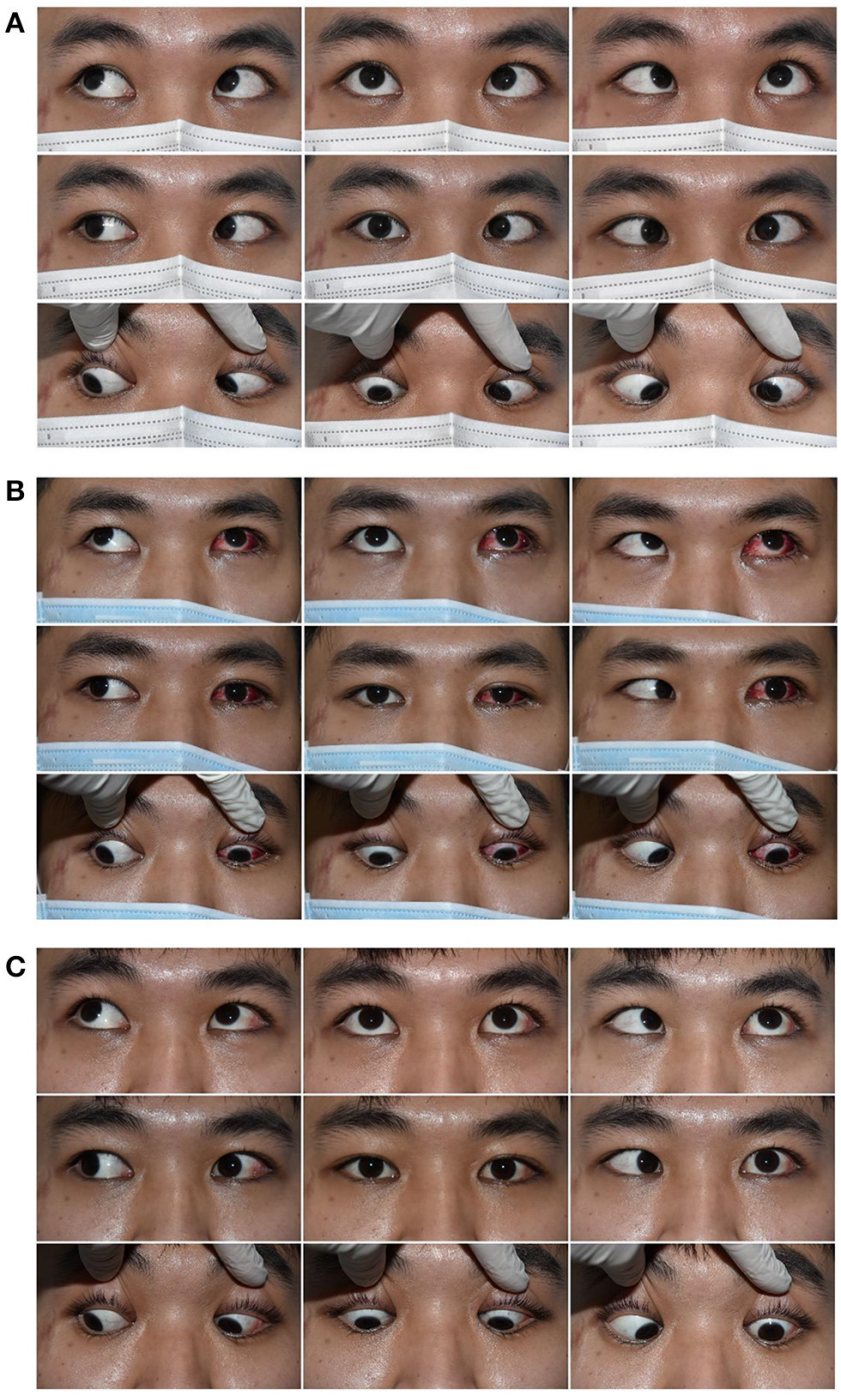
Typical case 1 showed the patient with abduction = −4 on the last visit. A 24-year-old male whose left eye deviated inward for 10 months after an injury resulting from a fall. He was diagnosed as a complete traumatic abducens nerve palsy and received a medial rectus recession of 10 mm and lateral rectus resection of 13 mm in his left eye while under general anesthesia. The FDT performed during surgery was negative. **(A)** The patient's 9-gaze eye position photo is shown preoperatively with left eye esotropia 50PD (LET = 50PD), abduction-6, adduction +1. **(B)** 1 day postoperatively with left eye exotropia 30PD (LXT = 30PD), abduction-2, adduction-4. **(C)** 2 months postoperatively with esotropia 3PD (ET = 3PD), abduction-4, adduction-2.

The left eye of a 24-year-old male deviated inward for 10 months after an injury resulting from a fall (Figures 3A–C). He was diagnosed with a complete traumatic abducens nerve palsy, and received a medial rectus recession of 10 mm and lateral rectus resection of 13 mm in his left eye while under general anesthesia. The FDT performed during surgery was negative. Figure 3A: The patient's 9-gaze eye position photo is shown preoperatively with left eye esotropia 50PD (LET = 50PD), abduction-6, adduction +1; Figure 3B: 1 days postoperatively with left eye exotropia 30PD (LXT = 30PD), abduction-2, adduction-4; Figure 3C: 2 months postoperatively with esotropia 3PD (ET = 3PD), abduction-4, adduction-2.

#### Case 2

**Figure 4:**
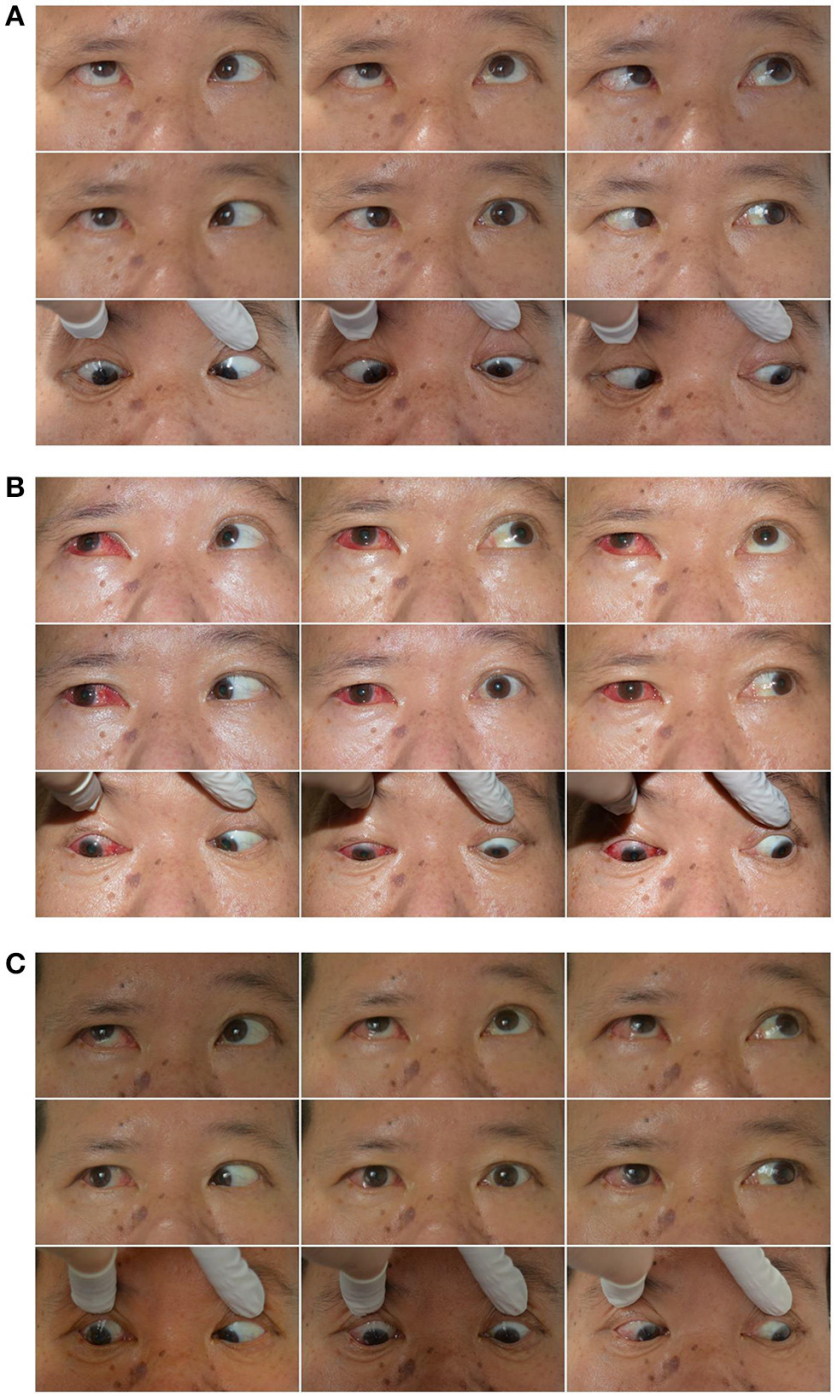
Typical case 2 showed the patient with abduction = −2 on the last visit. A 55-year-old male whose right eye deviated inward for one year after resection of a trigeminal neuroma. He was diagnosed as complete traumatic abducens nerve palsy and received a medial rectus recession of 8 mm and lateral resection of 11 mm in his right eye while under general anesthesia. The FDT performed during surgery was positive. **(A)** The patient's 9-gaze eye position photo is shown preoperatively with right eye esotropia 40PD (RET = 40PD), abduction-4, adduction normal. **(B)** 1 day postoperatively with right eye exotropia 20PD (RXT = 20 PD), abduction-1, adduction-4. **(C)** 2 months postoperatively with right eye exotropia 8PD (RXT = 8 PD), abduction-2, adduction-2.

The right eye of a 55-year-old male deviated inward for 1 year after resection of a trigeminal neuroma (Figures 4A–C). He was diagnosed with complete traumatic abducens nerve palsy, and received a medial rectus recession of 8 mm and lateral resection of 11 mm in his right eye while under general anesthesia. The FDT performed during surgery was positive. Figure 4A: The patient's 9-gaze eye position photo is shown preoperatively with right eye esotropia 40PD (RET = 40PD), abduction-4, adduction normal; Figure 4B: 1 days postoperatively with right eye exotropia 20PD (RXT = 20 PD), abduction-1, adduction-4; Figure 4C: 2 months postoperatively with right eye exotropia 8PD (RXT = 8 PD), abduction-2, adduction-2.

#### CASE 3

**Figure 5:**
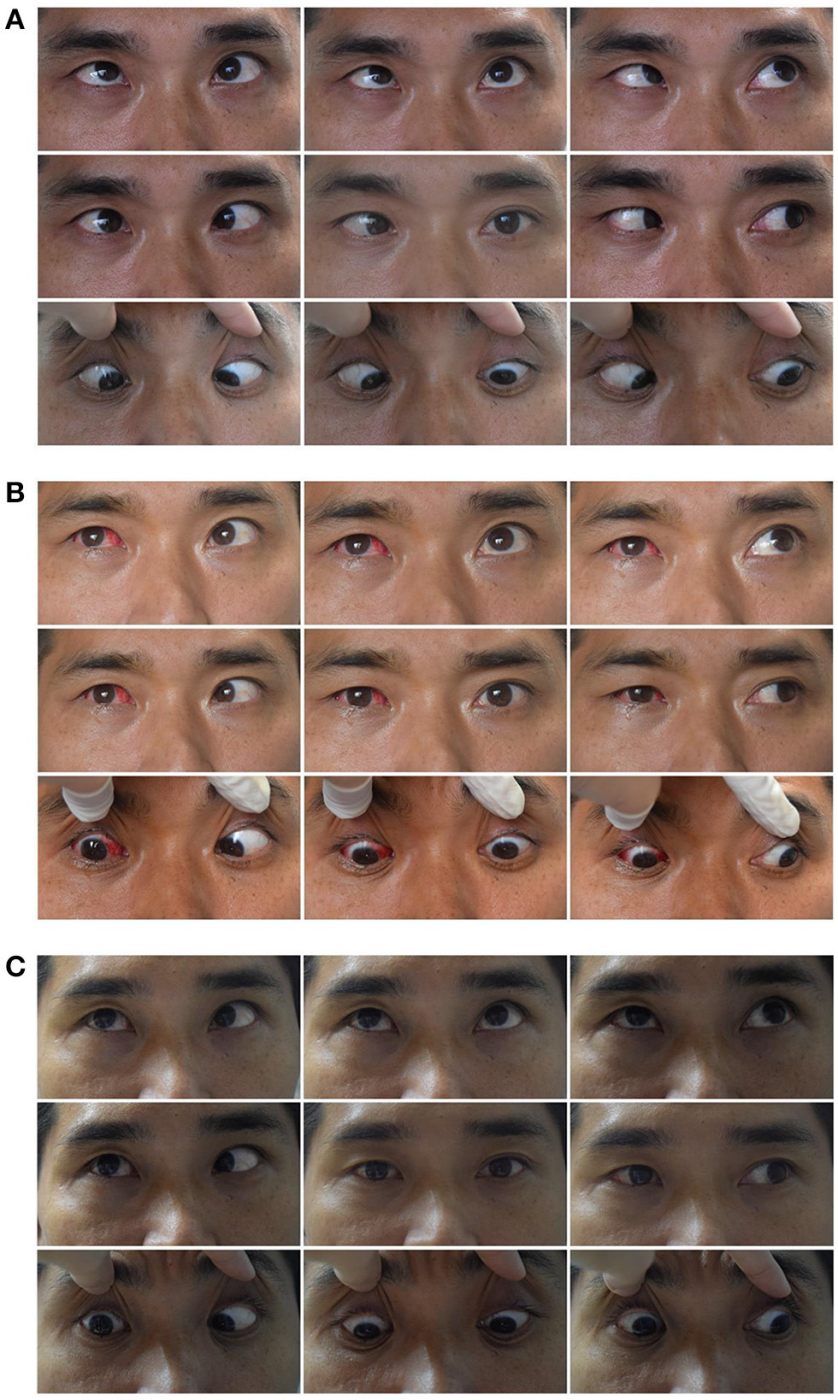
Typical case 3 showed the patient with abduction = 0 on the last visit. A 47-year-old male whose right eye deviated inward for 6 years. He was diagnosed as complete traumatic abducens nerve palsy and received a medial rectus recession of 10 mm and lateral rectus resection of 12 mm in his right eye while under general anesthesia. The FDT performed during surgery was (+++). **(A)** The patient's 9-gaze eye position photo is shown preoperatively with right eye esotropia 60PD (RET = 60PD), abduction-5, adduction + 1.5. **(B)** 1 day postoperatively with right eye exotropia 20PD (RXT = 20PD), abduction 0, adduction-4. **(C)** 2 months post-opeatively with right eye exotropia 7PD (RXT = 7PD), abduction 0, adduction-2.

The right eye of a 47-year-old male deviated inward for 6 years. He was diagnosed with complete traumatic abducens nerve palsy (Figures 5A-C), and received a medial rectus recession of 10 mm and lateral rectus resection of 12 mm in his right eye while under general anesthesia. The FDT performed during surgery was (+++). Figure 5A: The patient's 9-gaze eye position photo is shown preoperatively with right eye esotropia 60PD (RET = 60PD), abduction+5, adduction+1.5; Figure 5B: 1 days postoperatively with right eye exotropia 20PD (RXT = 20PD), abduction 0, adduction-4; Figure 5C: 2 months post-opeatively with right eye exotropia 7PD (RXT = 7PD), abduction 0, adduction-2.

## Discussion

Clinically, more than half of the patients with unilateral abducens nerve palsy can recover spontaneously, while an inability to abduct past midline at presentation and bilaterality were associated with a failure to recover (1). Relatively few of these patients require surgical treatment (15). Results from previous studies in patients receiving surgery with sample sizes greater than 30 (range: *n* = 33 to 71) (3, 13, 14, 16) have indicated that the average age of patients with abducens nerve palsy ranged from 20.4 to 43 years. In these patients, trauma was the leading cause of this condition, accounting for 54.5–63% of the patients, followed by 6.1–16.4% patients showing craniocerebral lesions. Ratios of males to females ranged from (0.97 :1) to (1.3 :1), while ratios of right to left eye ranged from (0.43 :1) to (1.35 :1). Similar to that reported in these studies, our current results indicate that the average age of patients with this condition was 37.8 ± 14.9 years, with women (27/36, 75%) being more susceptible. Trauma (47.2%, 17/36), followed by cerebral lesions (25%, 9/36) were the primary causes for this condition in these mostly middle-aged patients. There were no obvious differences between right and left eyes, but a significant gender difference was present.

In the present study, supramaximal recession–resection surgery without transposition procedure was sufficient for the treatment of complete sixth nerve palsy. In these patients, 82.6% achieved orthotropia, which was greater than that reported previously in patients receiving VRT or SRT surgery (Table 7). Excessive esotropia deviations resulting from complete sixth nerve dysfunction could be corrected with a simple recession–resection procedure, an effect which could be attributable to the following reasons. First, this procedure involved a 6–10 mm medial rectus “hang-back” recession, which could then result in a complete relaxation in the tonic contracture of the antagonist. The degree of ipsilateral medial rectus contracture is related to the efficacy of the tendon transposition procedure in sixth nerve palsy. A combination of medial rectus recession and partial transposition was reported to achieve a greater surgical corrective effect of 39 to 52 PD (22, 24). Second, lateral rectus resections of 8–14 mm generate pure mechanical tonic forces upon the paralyzed lateral rectus, although such procedures fail to regenerate the innervation needed to move the eyeball across the middle line with a slight over-correction in this cohort. “Muscle length adaption,” according to eye position and muscle stretch as described by Guyton, could play an important role in eye alignment (25). The medial rectus could be lengthened with the addition of sarcomeres to the medial and the lateral rectus could be shortened with the removal of sarcomeres from the shortened lateral rectus in this slightly exotropic eye position (26, 27). Finally, with the anatomical orientation of horizontal rectus muscles being in line with the visual axis, a maximal rotation force to maintain the eyeball straight in the primary position could be achieved than vertical muscle transposition procedure. Moreover, most adult patients with acquired abducens palsy have well-developed binocular vision. Retinal image disparity elicits fast fusional vergence which, in the short term, leads to vergence adaptation, producing a change in vergence tonus. As a result, these events stimulate muscle length adaptation over the long term, all of which reduce retinal image disparity. This three-level feedback process, as described by Guyton (25), completes the dynamic feedback system for maintenance of long-term binocular alignment after supramaximal horizontal rectus recession–resection surgery.

**Table 7:** Comparison of supramaximal–RR surgery with previous SRT and VRT procedures for abducens nerve palsy treatment.

**References**	**Year/Journal**	**Cases**	**Operation**	**Augmentation**	**Pre/post deviation (PD)**	**deviation corrected (PD)**	**Pre/post abduction deficition**	**Mean follow up (month)**	**Successful rate**	**Vertical** **deviation**
Rosenbaum et al. (17)	1989 Arch Ophthalmol	10	VRT	MRc/BT	55/5.4	49.6	-/20°	17.4	80%	–
Flanders et al. (5)	2001Can J ophthalmol	5	VRT	MRc/BT	52/1	66	−6/−1.7	21	100%	–
del Pilar González and Kraft (18)	2015J AAPOS	9	VRT	–	38.2/2.2	36	−4.1/−3.2	7.2	60%	–
		7	VRT	Resec 4 mm	48.9/2.5	46.4	−4.4/−2.7	6.7		
		9	VRT	Foster suture	60.3/19	41.3	−4.7/−3.3	9.5		
Lee and Lambert (9)	2017AMJ	8	VRT	Foster suture/BT/MRc	55.6/10.3	45.4	−4.5/−3.8	17.3	1.8additional surgery	–
		8	SRT	MRc/Foster suture	41.9/7.1	36.4	−4.6/−3.0	6.2	0 additional surgery	
Akar et al. (16)	2013Eye	47	VRT	Foster suture 64%BT	42.2/0.9	41.3	−3.9/−1.6	37	79%	–
Leiba et al. (19)	2010J AAPOS	22	VRT	BT 5u	38.1/7.8	30	−6/−2	44.2	59%	7
Yurdakul et al. (20)	2011opthalmic surgrey lasers imaging	17	VRT	MRc/FDT (+) Traction suture>65PD	64.1/9.6	54.5	−3.8/−2.5	18.7	76.5%	–
Kinori et al. (21)	2015J AAPOS	9	1/2VRT +Fostersuture	Plication augmentation BT/MRc, FDT (+)	70/1.5	83	−6.4/−2.7	28	67%	1
Couser et al. (22)	2012J AAPOS	9	1/2VRT+4 mm Resec	9cases MRc	43/6	39	−4/−3	7	80%	–
Patil-Chhab et al. (23)	2016 J AAPOS	13 (15 eyes)	SRT/Foster suture	MRc	55.4/9.9	46.7	−5/−3.1	5.2	69%	1
Mehendale et al. (8)	2012Arch ophthalmol	7	SRT/Foster suture	Adjusted MRc	53.5/16.8	36.5	−4.8/−3	10	71.4%	2
Current study		23	Ultra R-R	–	55.5/0.04	48	−6/−2	8.4	82.6%	9.6 (34.7%)

*VRT, Vertical rectus transposition; SRT, Superior rectus transposition; MRc, Medial rectus recession; BT, Botulinum; FDT, Forced duction tests; Ultra R-R, Supramaximal R-R operation; PD, Prism dioptre*.

In addition, abduction deficiencies also significantly improved, enabling a middle line cross in 65% (15/23) of the cases, with a few cases showing complete recovery. Patients with complete abducens palsy can also achieve a complete spontaneous recovery (1, 15). An inability to actively abduct across the midline was not taken to indicate an unrecovered palsy (3). The failure to recover within 6 months after palsy onset does not necessarily indicate that compensatory mechanisms of the nerve and muscles do not exist, it just means that they have yet to fully compensated or work. Supramaximal horizontal rectus recession–resection surgery may improve and/or partially restore abduction function by increasing the compensatory capacity of muscles and/or innervation through the following potential mechanisms. First, in the early post-operative period, a mechanical correction of eye position (mild overcorrection) produces muscle length adaptation, which regulates the number of muscle sarcomeres and muscle extensibility (26, 27). Second, a subset of patients developing clinical lateral rectus palsy may be due to lateral rectus superior compartment palsy, despite an intact lateral rectus inferior compartment (28). Residual lateral rectus function of contraction could not be detected until a supramaximal R-R procedure restored the ocular alignment. Supramaximal medial rectus recession has the effect of releasing tonic contraction and decreasing innervation impulses of the medial rectus, resulting in a reduced afterload of the lateral rectus during abduction. Furthermore, the enhanced mechanical tonic force resulting from supramaximal lateral resection increases the initial length of the muscle fiber sarcomeres, and muscle contraction forces are increased through the Frank–Starling law (29, 30). Finally, extraocular muscles may affect the functioning of extraocular muscle (EOM) stem cells, as activated by the maximal R-R procedure. Gene expression profiles of resected lateral recti in patients with complete lateral rectus paralysis were examined using microarray analysis and quantitative reversetranscription polymerase chain reaction (qRT-PCR) in our previous study (31). In that study, we found a decreased expression of Myogenic differentiation (MYOD) suggesting that differentiation processes of extraocular muscle satellite cells (EOMSCs) were inhibited. On the other hand, the high expression levels of the transcription factor Paired box 7 (PAX7), SIX homeobox 1/4 (SIX1/4), and Myogenin (MYOG), suggest that these EOMSCs were showing an effective potential for differentiation. The stimulation resulting from muscle surgery may induce EOMSCs to differentiate and thus produce a compensatory restoration of abduction.

When comparing the clinical characteristics of patients who were able to cross the midline with those who were not, we found that 93.3% (14/15) of the patients who were able to abduct and cross the midline post-operatively showed a mild resistance in their pre-operative FDTs, while only 25% (2/8) of patients who were unable to cross the midline post-operatively had resistance in their pre-operative FDTs. Moreover, this latter group also demonstrated greater insufficiencies of external rotation before surgery. Interestingly, no differences were present between these two groups with regard to pre-operative esodeviation, which indicated that the degree of esodeviation may not serve as a good index for evaluating the degree of lateral rectus function.

Based on our observations, all supramaximal recession–resection procedures resulted in overcorrection and adduction deficiencies as assessed on the first day after surgery. However, this overcorrection regressed, and adduction efficiency gradually improved and stabilized when evaluated 2 months after surgery. Mean overcorrections on the first day post-operatively was −11.5 ± 11.0 PD, while inward back deviations of 12 PD were observed from the first day post-operatively to their last visit. Despite a decrease in adduction function after surgery, mean horizontal duction range changed from 3.4 ± 1.0 units pre-operatively to 3.8 ± 1.4 units on their last visit, but the difference was not statistically significant. These findings demonstrate that improvements in alignment and duction did not decrease the duction range of the affected eye. Therefore, in supramaximal recession–resection procedures with an adjusted suture technique as performed under local anesthesia, the intraoperative eye position should be directed to an exodeviation of 12 PD, which could improve the long-term orthotopic probability.

Surgical dose-effect coefficients, the deviation corrected per mm in the R-R procedure, were much smaller in complete abducens palsy than in concomitant esotropia. Such an effect results in the failure of conventional dosage designed for recession–resection procedures in complete abducens palsy. The small dose-effect coefficients were determined by low or absent tonic forces of paralyzed lateral rectus, which necessitate more recession to reduce medial rectus contracture and/or antagonistic tonic forces. Although etiology, disease duration, pre-operative deviation, and adduction function were all associated with dose-effect coefficients in the univariate analysis, only pre-operative deviation was an independent factor of surgical dose-effects as based on multifactorial analysis. These findings imply that the supramaximal R-R surgery is a self-adjusting surgery and the same surgical dosage can correct esodeviations in patients with larger esotropia, as were evident in cases with esotropia >50 PD.

Bagheri (14) assessed the correlation between baseline eye deviations and post-operative improvement. They found that the greater the primary deviation, the better the response to primary surgery procedures, including not only horizontal rectus surgery without transposition but also vertical rectus transposition procedures and botulinum toxin injection, with the recession–resection procedure producing the steepest slope. This self-adjusting feature could likely be due to the excessive horizontal rectus recession and resection, which exceeds the range of the muscle's elastic modulus. In addition, when contraction of the medial rectus muscle is sufficiently diminished by a large recession along with a diminished or lost paralytic lateral rectus muscle force, orbital anatomic structures including the tapered structures of the bony orbit, the extraocular muscular cone, the orbital fat, and the fascial pulley system could exert a natural role in maintaining the eye in the primary position.

Recession–resection procedures avoid the potential complications of vertical rectus transpositions, including new vertical deviations in the primary position, new torsion and anterior segment ischemia, as reported in the literature (5, 32, 33). In the current study, vertical deviations were comparable and slightly increased from 26.1 to 34.7% after maximal recession–resection surgery. Four cases of vertical deviation were eliminated and 6 cases occurred after surgery. Hypertropia is commonly associated with isolated, unilateral abducens nerve palsy. The reported incidence ranged from 19 to 61%, with deviations ranging from 4 to 16 PD, depending on the measurement method employed (28, 34, 35). Etiology of this hypertropia has been controversial, but has been hypothesized to involve a physiological hyperphoria unmasked by a horizontal, compartmental paralysis of the superior or inferior zones of the affected lateral rectus muscle (36). As the recession–resection procedure did not change the direction of the horizontal muscle force, there were no significant pre- vs. post-surgery differences in the proportion and degree of vertical deviation.

There are some inherent limitations in our study. It was a retrospective clinical study with no controls. All clinical data were from a single center and all surgeries were performed by one surgeon. Complete abducens palsies were diagnosed only based on abductions that failed to cross the middle line, although this method is simpler to assess and commonly used in clinical settings. Follow-up periods were relatively short. It will be necessary to prospectively evaluate and compare the supramaximal horizontal rectus recession–resection procedure with vertical rectus transposition in complete abducens palsy as assessed in randomized clinical trial studies.

Despite these limitations this study represents the first investigation directed at evaluating the clinical effects of supramaximal horizontal rectus recession–resection procedures as applied for the treatment of complete abducens palsy. The findings of this study offer important, new perspectives as related to complete abducens palsy.

## Data Availability Statement

The original contributions presented in the study are included in the article/supplementary material, further inquiries can be directed to the corresponding author/s.

## Ethics Statement

The studies involving human participants were reviewed and approved by the Research Ethics Board of the Zhongshan Ophthalmic Center (ZOC), Sun Yat-sen University, China; Institutional approval (Approval No: 2021KYPJ050). Written informed consent to participate in this study was provided by the participants' legal guardian/next of kin. Written informed consent was obtained from the individual (s), and minor (s)' legal guardian/next of kin, for the publication of any potentially identifiable images or data included in this article.

## Author Contributions

ZW, LF, and JY were involved in the methodology. JY was involved in the validation. ZW, LF, TS, and XQ performed the formal analysis. ZW and JY were in charge of the resources. ZW and LF were responsible for data curation. ZW, LF, and TS wrote the original draft. TS, XQ, XY, and JY were involved in reviewing and editing the draft. HS and JY supervised the study and took care of project administration. JY was involved in funding acquisition. All authors contributed to the article and approved the submitted version.

## Funding

This work was supported by the National Natural Science Foundation of China (Grant Number: 81670885). The funders had no role in the study design, data collection and analysis, decision to publish, or preparation of the manuscript. No additional external funding was received for this study.

## Conflict of Interest

The authors declare that the research was conducted in the absence of any commercial or financial relationships that could be construed as a potential conflict of interest.

## Publisher's Note

All claims expressed in this article are solely those of the authors and do not necessarily represent those of their affiliated organizations, or those of the publisher, the editors and the reviewers. Any product that may be evaluated in this article, or claim that may be made by its manufacturer, is not guaranteed or endorsed by the publisher.
